# Alveolar Abscess

**Published:** 1895-10

**Authors:** 


ARTICLE VII.

### ALVEOLAR ABSCESS.

Dr. N. Pearson says: "We do sometimes meet a case where there is fistula without an apical opening, or if there is one we are not able to find it, and no remedy can be forced through without drilling. In such a case we will find a broken Morey drill made sharp by stoning two sides to a cutting edge, or by using the regular Morey cutter very effective, using care in choice of roots to be opened, or such as are accessible to straight drills, and open them only at the apex. In dealing with roots of this kind I have been able especially in inferior teeth, to force a passage by warming gutta-percha and pushing it into the chamber and canals with the tip of the forefinger or thumb, or an instrument quite as large as the cavity; thus making a strong piston force.

"In still other cases where we have a curved root, and in consequence a liability to make a side issue or other good reasons for not piercing the apex, by using a syringe with hydrogen peroxide and getting as near the seat of the abscess as possible through the external opening, and a very mild pressure on the piston, we may be able to persuade the contents of the sac to imbibe sufficient of the antiseptic to affect a cure. There is still another way of getting at an abscess, which for some reasons we are not inclined to apply other means of remedying, and this is through the process: By using cocain or other local or general anæsthetic, or by proceeding without these aids. The operation is not very painful, and is soon done with. In doing this a sharp fissure drill in the engine may be rapidly run over the end of the root, completely severing the connection between root and sac, followed by any good antiseptic and soothing remedy thought best. Cut, and in a few days all trace of abscess disappears."—*Dominion Journal.*

---

